# Fish as Food

**Published:** 1867-12

**Authors:** 


### FISH AS FOOD.

He next spoke of fish, as an article of food, illustrating his remarks by boning and skinning a perch:

"A fish boiled in water alone is not inferior to one sewed in cloth and boiled. There is no better fish in the world for frying than our American smelt. The speaker said lobster was very nutritive, but heavy, and should never be eaten at supper. Referring to oysters, he said that they should never be fried in butter, and should be served hot. Potatoes should be steamed and not boiled; and he cautioned his class against using water that had been standing on the stove, as the gases and alkali are all absorbed in the first boil. Vegetables boiled in such water are inferior. Dried Lima beans are very bad for people living in cities. Some Ladies will not boil cabbage because of the smell. This could be avoided by simply putting a piece of charcoal in the pot. Lentils are far better for eating than either peas or beans. Many persons, however, suppose them dearer than peas or beans, not knowing that they swell three or four times their size when soaked before cooking. He said that, if more dandelions and chicory were eaten in the spring, there would be less need of medicine and instanced a case of a family who spend $30 a month for medicine, and did not see why they were sick. Referring to radishes, the Professor said that there was a substance in the center leaves of each root, which, if eaten, would help their digestion. Every family should be well supplied with sorrel and tomatoes the year round, these vegetables being the greatest neutralizers of acrid substances. Water-cresses should never be eaten over twice in a week. They contain much sulphur, and are the greatest anti-scorbutic known. The speaker then instructed his students in the art of making coffee, and, in conclusion, advised the use of gauze strainers instead of tin perforators.

